# Supplementary material for: Calcitriol Modulates Age-dependent Drug Response in Paired Patient-derived Normal and Tumor Colorectal Organoids
Source: Int J Biol Sci. 2026 Jul 30;22(13):7003–17. doi: 10.7150/ijbs.131639 (PMC13540450; doi:10.7150/ijbs.131639)
Supplement: Supplementary file 1 [file ijbsv22p7003s1.pdf]

Table S1: Composition of culture media for colorectal normal and tumor PDOs.

Reagent	Concentration	Source	Normal medium	Tumor medium
Advanced DMEM/F12		Thermo Fisher Scientific	50%	100%
HEPES	10 mM	Thermo Fisher Scientific	+	+
Glutamax	10 mM	Thermo Fisher Scientific	+	+
B27 supplement	1X	Thermo Fisher Scientific	+	+
N2 supplement	1X	Thermo Fisher Scientific	+	+
Nicotinamide	10 mM	Sigma-Aldrich	+	-
N-acetyl-L-cysteine	1 mM	Sigma-Aldrich	+	+
Primocin	0.1 μg/mL	Invivogen	+	+
L-WNR (Wnt3A, Noggin, RSPO3) conditioned medium		Custom made (Ref. #41)	50%	-
RSPO1	1 μg/mL	Sinobiological	+	-
PGE_2	0.02 μM	Sigma-Aldrich	+	+
EGF	50 ng/mL	Peprotech	+	+
Gastrin	1 μg/mL	Tocris	+	+
Noggin	0.1 μg/mL	Sinobiological	+	+
Y-27632*	10 μM	Tocris	+	+
LY2157299	1 μM	Axon-Medchem	+	+
SB202190	10 μM	Sigma-Aldrich	+	+

* Only in single cell suspensions

Table S2: Clinicopathological characteristics of CRC patients whose biopsies were used to establish normal and tumor organoid cultures

Patient number	Sex	Age (years)	Classification	Tumor localization	T[a]	N[b]	M[c]	Drug assay (passage)		RNA-seq (passage)	
								Normal	Tumor	Normal	Tumor
30	Male	81	LO-CRC	Left colon	4	1	0	P7	P40	P12	P30
48	Male	83	LO-CRC	Left colon	4	1	0	P9	P50	-	-
53	Male	81	LO-CRC	Left colon	3	0	0	-	-	P3	P2
57	Male	86	LO-CRC	Left colon	4	1	0	-	-	P1	P8
66	Male	76	LO-CRC	Left colon	3	0	0	P8	P35	P3	P5
73	Female	40	EO-CRC	Left colon	3	1	0	P11	P18	P7	P6
74	Male	84	LO-CRC	Left colon	3	0	0	-	-	P7	P3
89	Male	41	EO-CRC	Right colon	4	0	1	P15	P10	P11	P10
110	Male	57	LO-CRC	Left colon	3	0	0	P9	P6	-	-
113	Male	71	LO-CRC	Left colon	3	0	0	P9	P8	-	-
120	Male	45	EO-CRC	Left colon	3	0	0	P2	P13	P3	P5
122	Male	86	LO-CRC	Left colon	4	0	0	-	-	P7	P11
175	Female	46	EO-CRC	Rectum	4	2	0	P12	P11	-	-
183	Male	49	EO-CRC	Left colon	1	1	0	-	-	P3	P5
200	Male	48	EO-CRC	Left colon	4	1	0	P7	P8	P4	P5

T[a]: Size or direct extent of the primary tumor; N[b]: degree of spread to regional lymph nodes; M[c]: presence of distant metastasis (www.uicc.org/resources/tnm).
P: Indicates the organoid passage at which each experiment was performed
-: Indicates that the corresponding PDO was not used for that analysis

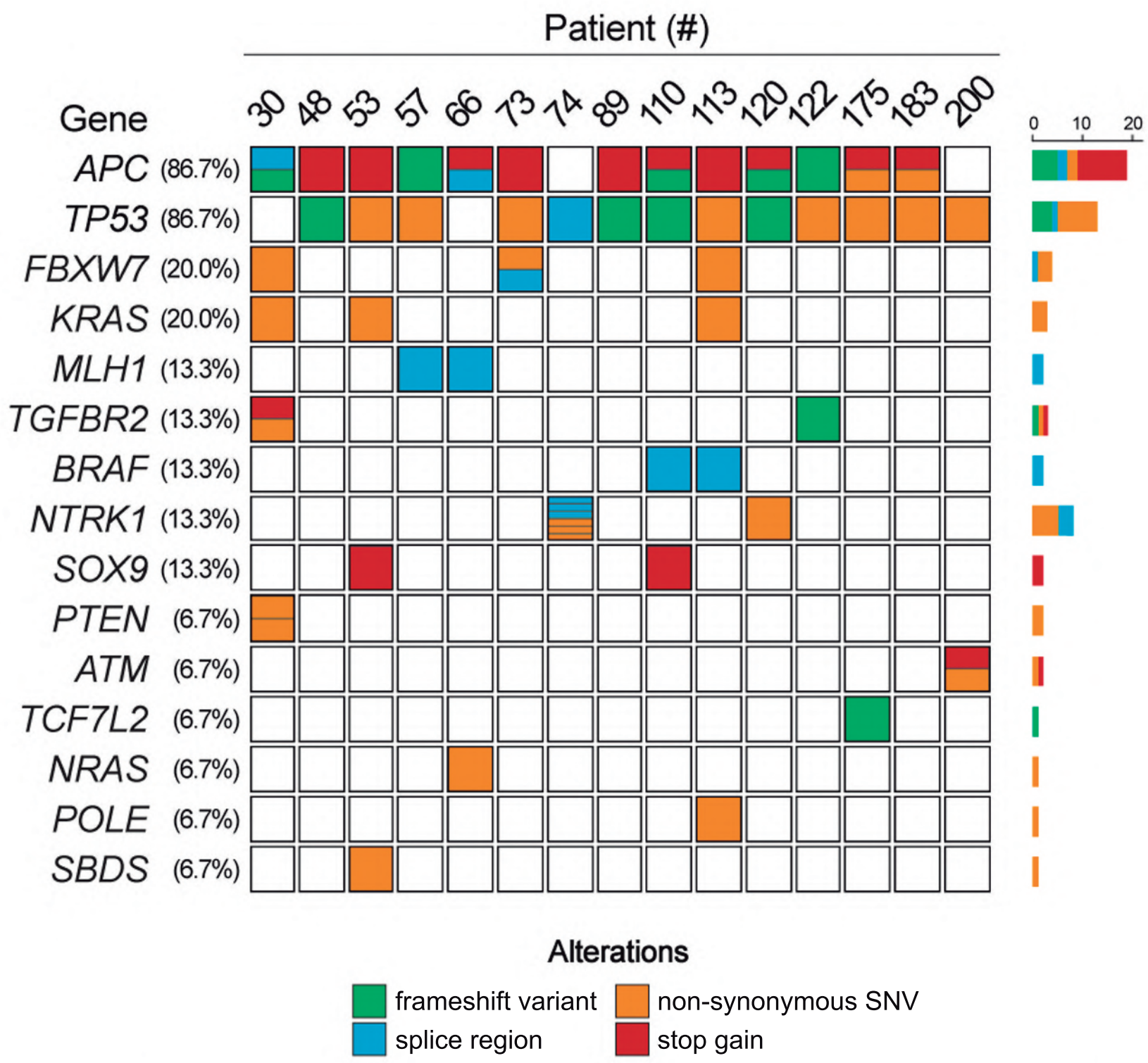

Figure S1: Somatic alterations found in tumor PDOs. Oncoprint representing mutations found in the tumor PDOs used for drug cytotoxicity assays and/or RNA-seq assays (n = 15 PDOs). Columns and rows respectively represent patients and genes (ordered by mutation frequency). Color indicates mutation type. Bar plot on the right displays the number of alterations identified for each gene. SNV, single nucleotide variant.

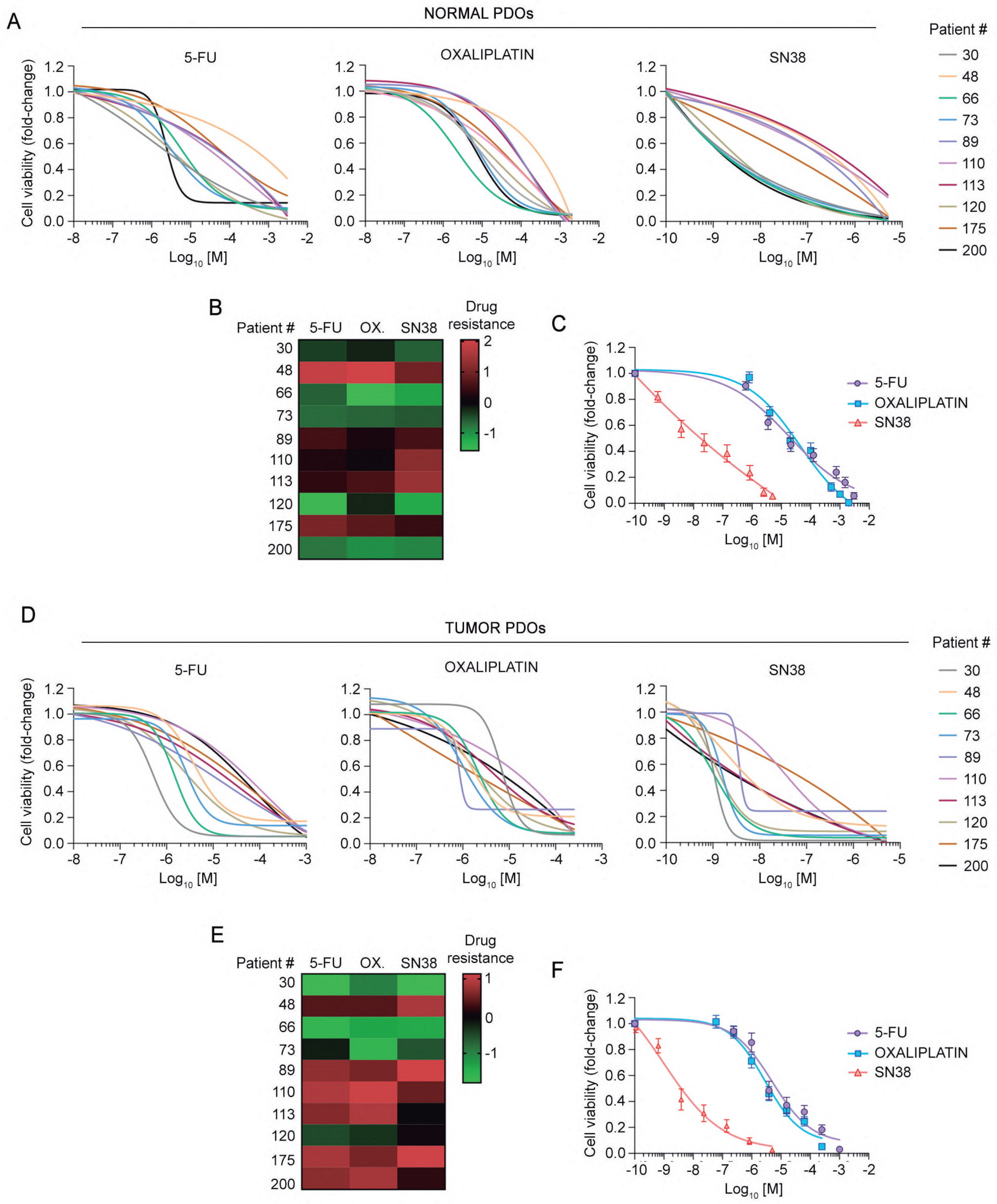

Figure S2: Response of colorectal normal and tumor PDOs to 5-FU, oxaliplatin and SN38. (A) Dose-response curves of 10 normal PDOs treated with 5-FU, oxaliplatin or SN38. Each line represents a patient. (B) Heatmap illustrating the z-score of drug resistance of normal PDOs from (A) for each drug treatment. (C) Dose-response curves (mean values ± SEM) of normal PDOs used in (A). (D) Dose-response curves of 10 tumor PDOs treated with 5-FU, oxaliplatin or SN38. Each line represents a patient. (E) Heatmap illustrating the z-score of drug resistance of tumor PDOs from (D) for each drug treatment. (F) Dose-response curves (mean values ± SEM) of tumor PDOs used in (D).

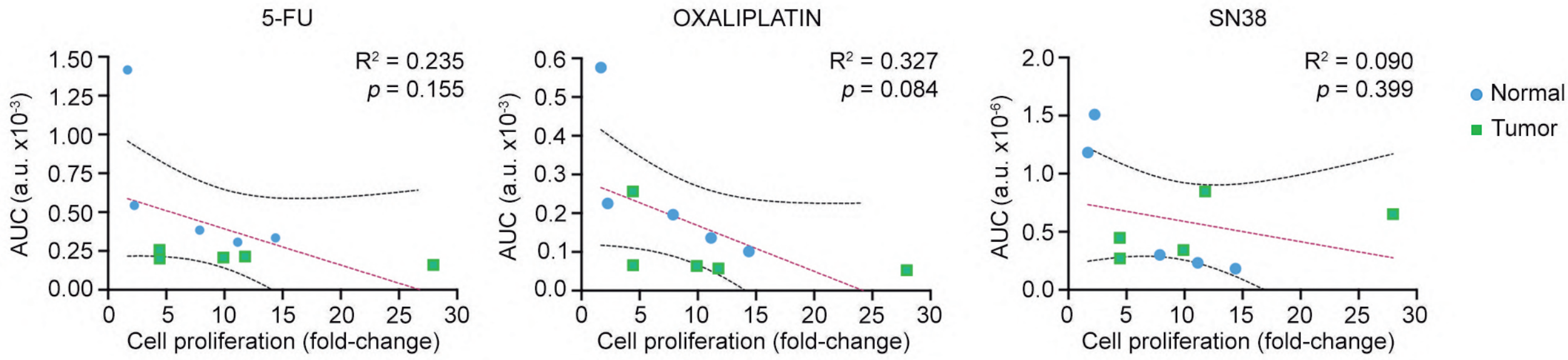

Figure S3: Relationship between proliferation rates and drug cytotoxicity in colorectal normal and tumor PDOs. Scattergrams showing the correlations between cell proliferation rates (assessed after 4 days in culture) and AUC values derived from the drug assays shown in Figure S2A and S2D. Each dot represents an individual normal (blue) or tumor (green) PDO. Correlation was evaluated by Pearson's test (n = 10 PDOs).

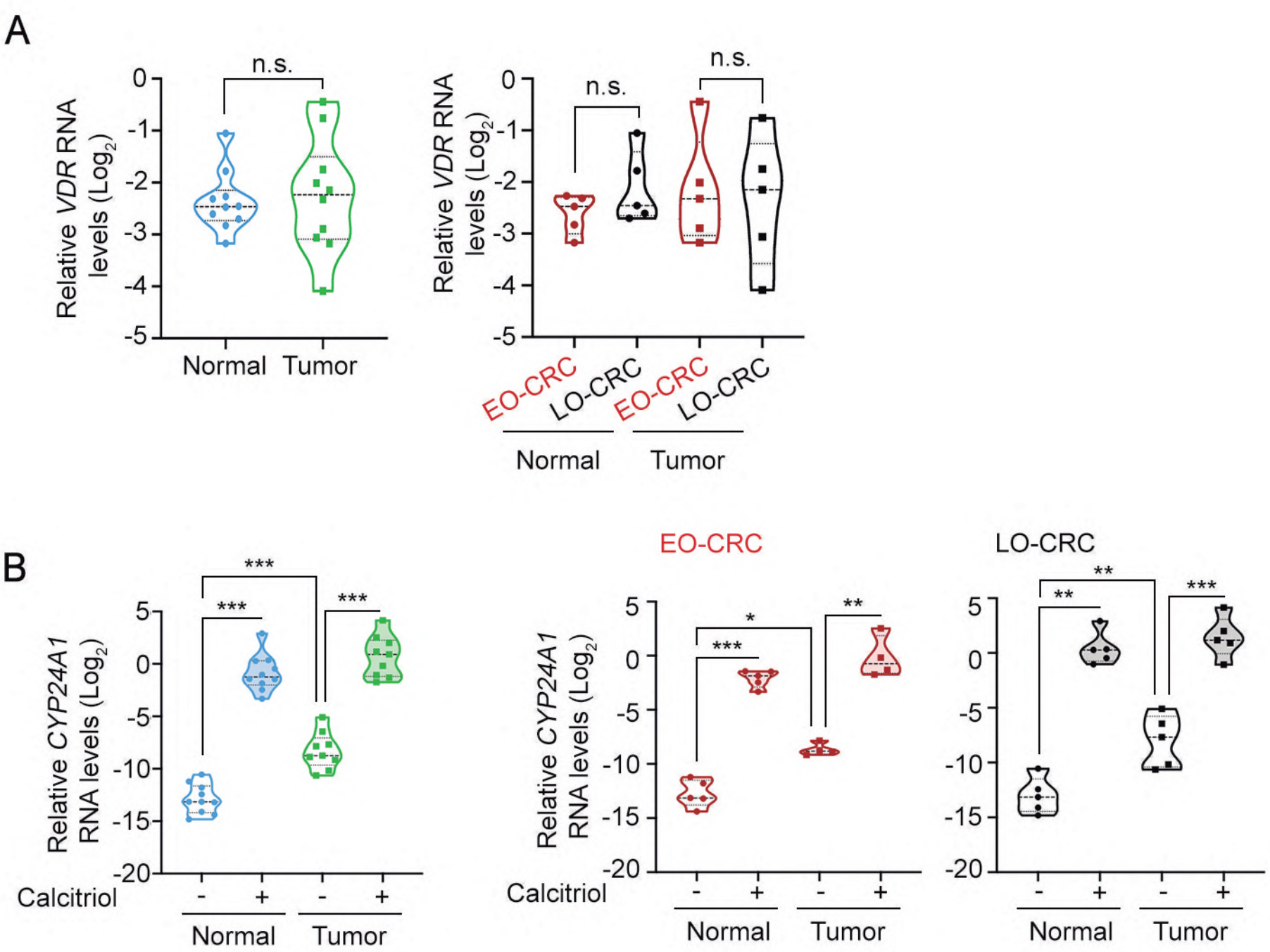

Figure S4: Colorectal normal and tumor PDOs respond to calcitriol. (A) Violin graphs of *VDR* RNA levels in overall (left panel) and age-grouped (right panel) normal and tumor PDOs (n = 10 paired PDOs; EO-CRC n = 5, LO-CRC n = 5). Statistical analysis for paired comparisons (normal *vs.* tumor) was performed using two-tailed paired *t*-test, and comparisons between EO-CRC and LO-CRC groups were performed using two-tailed unpaired *t*-test. (B) Violin graphs of *CYP24A1* RNA levels in overall (left panel) and age-grouped (middle and right panels) normal and tumor PDOs treated for 96 h with 100 nM calcitriol or vehicle (ethanol) (n = 10 paired PDOs; EO-CRC n = 5, LO-CRC n = 5). One-way ANOVA followed by Sidak's post-test was applied for multiple comparisons.

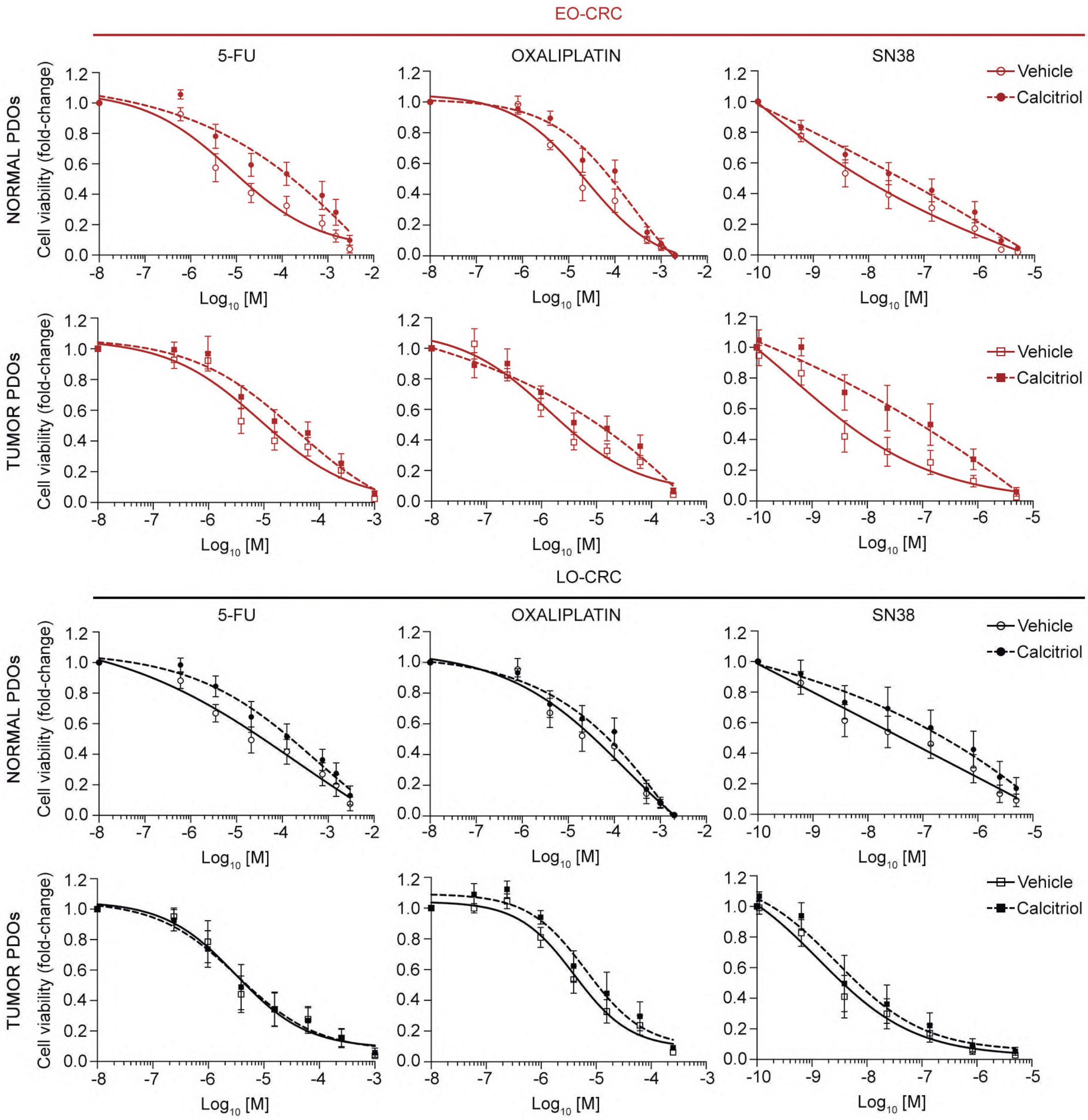

Figure S5: Effect of calcitriol on drug cytotoxicity in EO-CRC and LO-CRC normal and tumor PDOs. Dose-response curves of normal and tumor organoids from EO-CRC and LO-CRC patients treated with 5-FU, oxaliplatin or SN38 in the presence of 100 nM calcitriol or vehicle (ethanol) for 96 h. Mean values ± SEM are shown (n = 5 PDOs per group).
